# Effect of Fu Yan Qing prescription on pelvic effusion, mass absorption and microenvironment of pelvic blood stasis in patients with sequelae of pelvic inflammatory disease of accumulation of dampness heat and blood stasis type

**DOI:** 10.12669/pjms.38.5.4641

**Published:** 2022

**Authors:** Sujuan Zhang, Lijin Yuan, Yunliang Wang, Zhe Sun

**Affiliations:** 1 Sujuan Zhang, Department of Outpatient, Baoding No.1 Central Hospital, Baoding, Hebei, 071000, China; 2 Lijin Yuan, Department of Obstetrics, East Hospital of Baoding No.1 Central Hospital, Baoding, Hebei, 071000, China; 3 Yunliang Wang, Medicine Oncology Clinic, Baoding No.1 Central Hospital, Baoding, Hebei, 071000, China; 4 Zhe Sun Department of Outpatient, West Hospital of Baoding No.1 Central Hospital , Baoding, Hebei, 071000, China

**Keywords:** Fu Yan Qing decoction, Accumulation of dampness heat and blood stasis, Sequelae of pelvic inflammatory disease, Pelvic effusion, Mass absorption, Pelvic blood stasis microenvironment

## Abstract

**Objective:**

To investigate the effect of Fu Yan Qing prescription on sequelae of pelvic inflammatory disease of accumulation of dampness heat and blood stasis type.

**Methods:**

Total 80 patients with sequelae of sequelae of pelvic inflammatory disease of accumulation of dampness heat and blood stasis type were admitted to Baoding No.1 Central Hospital from December, 2018 to April, 2020 and divided into two groups according to the random number table, with 40 cases in each group. Patients in the control group were treated with conventional western medicine, while patients in the observation group were treated with Fu Yan Qing prescription orally. The clinical efficacy, the changes of traditional Chinese medicine (TCM) syndromes, local sign scores, visual analog scale (VAS) of pain, pelvic mass size, pelvic fluid volume and uterine blood flow parameters of the two groups before and after treatment were observed and compared, and the safety of the two groups was evaluated.

**Results:**

The total efficacy after treatment in the observation group was 87.5%, which was significantly higher than that of 67.5% in the control group (*p<*0.05). The TCM syndrome scores, local signs scores, pain scores, size of pelvic mass and pelvic effusion in both groups decreased significantly after treatment (*p<*0.05), PSV indexes of the two groups were significantly increased after treatment (*p<*0.05), and these changes were even more pronounced in the observation group (*p<*0.05). Compared with before treatment, PI and RI indexes of the observation group were significantly decreased after treatment (*p*<0.05). The observation group experienced an adverse reaction in 7.5% cases considerably lower than the 27.5% of the control group (p<0.05).

**Conclusion:**

Fu Yan Qing prescription is a safe and reliable treatment for patients with sequelae of pelvic inflammatory disease of accumulation of dampness heat and blood stasis type. It is worth promotion in clinical practice.

## INTRODUCTION

Pelvic inflammatory disease (PID) mainly refers to infectious diseases that are present in the upper genital tract of women, including salpingitis, ovarian cysts, and endometritis. Sequelae of pelvic inflammatory disease (SPID) can be attributed to improper or untimely PID treatment, and repeated prolonged course of disease. Its pathological changes are mainly characterized by tissue destruction, scar formation, extensive hyperplasia and tissue adhesion. Patients with SPID present with recurrent lower abdomen pain, lumbosacral soreness, lower abdominal distension and abnormal leucorrhea, which can be complicated with ectopic pregnancy, chronic pelvic pain and infertility in the long term, seriously affecting the quality of life and reproductive health of female patients.^1^,^2^

In Western medicine, broad-spectrum antibiotics are currently being given priority as a treatment for SPID. However, antibiotic resistance has been significantly increased due to the long course of SPID, frequent relapses, and long-term repeated medication, which is not conducive to controlling the development of SPID.^3^,^4^ SPID can be classified into the categories of “abdominal mass”, “leucorrhea” and “abdominal pain” in traditional Chinese medicine, with its main pathogenic factors being damp heat and excessive pathogen.^5^ The key to the lingering and unhealed condition of SPID lies in the internal stasis of dampness and heat, which can stream into the Chong and Ren channels as well as uterus, and then evils of Qi and blood blended to attack the body, with the blood stasis in lower jiao, resulting in unsmooth circulation of the blood in the uterus and the pathogenesis of dampness heat and blood stasis.^6^

In this study, Fu Yan Qing decoction, which has the characteristics of clearing heat and dehumidifying, promoting blood circulation and removing blood stasis, was designed based on the pathogenesis characteristics of “damp-heat stasis” of SPID. Emphasis was placed on observing the effects of Fu Yan Qing decoction on the TCM syndrome score, pelvic effusion, mass absorption and pelvic blood stasis microenvironment in patients with SPID.

## METHODS

A total of 80 patients with SPID admitted to Baoding No.1 Central Hospital from December 2018 to April 2020 were enrolled as the observation subjects. All the subjects were in line with the following diagnostic criteria and inclusion criteria, and no cases dropped out of the clinical trial during the course of treatment.

### Ethical approval:

The study was approved by the Institutional Ethics Committee of Baoding No.1 Central Hospital on January 12, 2019(No.: 1941ZF065), and written informed consent was obtained from all participants

### Diagnostic criteria:

Patients were diagnosed as obstetrics and gynecology disease in Western medicine diagnosis according to the guideline of pelvic inflammatory disease 7: It included patients with a history of pelvic inflammatory disease; Patients whose main clinical manifestations are chronic pelvic pain, menstrual disorders, abnormal leucorrhea, infertility, and occasionally fatigue, low fever, mental weakness and other systemic symptoms; The patient was found to have limited or tenderness in the uterus or uterine appendices during the gynecological examination; Patients who have a significant increase in the number of white blood cells via vaginal secretions examination and have been detected with pathogenic bacteria; Patients with tubal effusion, tubal and ovary mass and pelvic effusion observed by ultrasound examination. Patients who are dialectically diagnosed as suffering from dampness heat and blood stasis syndrome based on the SPID dialectical standards drawn up in the dialectical criteria of pelvic damp-heat stasis syndrome in TCM^8^,^9^: The main symptoms are lumbosacral pain, pain in the lower abdomen, fixation of the pain, increased amount of lower band, yellow, yellow-green or yellow-white color, sticky texture; The secondary symptoms are prolonged menstrual period, increased menstrual volume, irregular vaginal bleeding, entrained blood clots, epigastric stay, low fever, abdominal pain after fatigue or increased menstrual period, constipation, yellow and red urine; dark red tongue, stasis on the edge of the tongue point, yellow and greasy tongue coating, thready and slippery pulse.

### Inclusion criteria:

- Patients who meet the above-mentioned diagnosis criteria of “SPID disease” in Western medicine and Chinese medicine, as well as the TCM syndrome differentiation criteria of “dampness heat and blood stasis syndrome”;
- Patients aged between 20 and 45 years;
- Patients with a history of sexual life;
- Patients who have signed the written informed consent for this clinical trial.

### Exclusion criteria: -

- Patients with combined pelvic space occupying disease and pelvic organic disease;
- Patients with renal, liver and heart dysfunction and autoimmune deficiency;
- Patients who are pregnant or have a pregnancy plan in the near future;
- Female patients who are breastfeeding;
- Patients with mental illness or severe mental retardation;
- Patients with allergies or allergic reactions to the drugs in this study.

All the 80 patients were divided into two groups according to the random number table: the observation group and the control group, with 40 cases in each group. Patients in the control group ranged in age from 22-44 years old, with an average of (33.16±4.37) years old. The shortest course of disease was 4 months and the longest was 32 months, with an average of (8.15±1.58) months. Patients in the observation group ranged in age from 23-45 years old, with an average of (33.21±4.29) years old. The shortest course of disease was four months and the longest was 33 months, with an average of (8.19±1.55) months. There was no statistically significant difference in the above-mentioned data (mean age, mean course of disease) between the two groups (*p*>0.05).

Patients in the control group received an intravenous drip of 0.8 g tinidazole and glucose injection (Sichuan Qili Pharmaceutical Co., Ltd., State Drug Approval No. H10970277, Strength: 100 ml: tinidazole 0.4 g and glucose 5 g), qd; and 0.4g levofloxacin hydrochloride injection (Yangzijiang Pharmaceutical Group Co., Ltd., State Drug Approval No. H20060337, Strength: 2 ml:0.2 g), intravenously once every 12 hour. After remission, the patients were treated with oral tinidazole tablets (Zhejiang Hangkang Pharmaceutical Co., Ltd., National Medicine Zhunzi H33020324, Strength: 0.5 g), 1.0g a day, qd; and oral levofloxacin hydrochloride tablets (Zhejiang Jingxin Pharmaceutical Co., Ltd., State Drug Approval No. H19990060, Strength: 0.1 g), 0.2 g each time, bid. A total of three cycles of treatment were carried out with two weeks as a cycle.

### Observation Group:

Patients in the observation group were given oral treatment with self-made Fu Yan Qing decoction. Ingredients: 15 g each of Radix Paeoniae Rubra, Salvia miltiorrhiza, Fructus Ligustri Lucidi; 12 g each of Patrinia villosa Juss, Forsythiae Forsythiae, Rhizoma corydalis, Radix Dipsaci, Rhizoma Smilacis Glabrae; 10g each of Honeysuckle, Plantain Seed and Rhizoma Cyperi; 8 g of Rhizoma Curcumae. The above pharmaceutical ingredients were prepared into decoction-free granules according to the prescriptions of Baoding No.1 Central Hospital . The granules should be dissolved with 200 ml of boiled water before each dose, and taken twice in the morning and evening. A total of three cycles of treatment were carried out with two weeks as a cycle.

### Observation Indexes:

Scores of TCM syndromes and signs: patients with lower abdominal pain or tingling, lumbosacral pain and leucorrhea before and after treatment, and score the local signs of patients with uterine movement restricted tenderness, thickening and tenderness in the adnexal area, adnexal mass, tenderness, uterosacral ligament before and after treatment.The VAS score was used to evaluate the improvement of pain before and after treatment, with 0 as painless and 10 as intolerable pain. The higher the score, the more severe the pain.Patients underwent vaginal color Doppler ultrasound examination three to seven days after menstruation before and after treatment, and the size of pelvic mass and the volume of pelvic effusion were detected and recorded.

### Uterine blood flow parameters:

Patients underwent vaginal color Doppler ultrasound examination three to seven days after menstruation before and after treatment, and their peak systolic velocity (PSV), pulse index (PI) and resistance index (RI) of the uterine and ovarian arteries were detected.

### Safety Analysis:

The incidence of ADR in the two groups during treatment was compared and statistically analyzed.

### Efficacy Criteria:

Patients whose main symptoms such as lumboseal pain and lower abdominal pain were completely disappeared, gynecological examination indexes and physical and chemical examination indexes all returned to normal, and whose symptom scores decreased by more than 95% should be regarded as cured; Patients whose lumbosacral distend pain and lower abdominal pain were significantly relieved, gynecological examination indexes and physical and chemical examination indexes were significantly improved, and whose symptom scores decreased by 70%-94% should be regarded as markedly effective; Patients whose lumbosacral distend pain and lower abdominal pain were relieved to some extent, gynecological examination indexes and physical and chemical examination indexes were improved to some extent, and whose symptom score decreased by 30%-69% should also be regarded as effective; Patients whose lumbosacral distend pain and lower abdominal pain were not relieved or even worsened, gynecological examination indexes and physical and chemical examination indexes were not improved, and whose symptom scores decreased by less than 30% should be regarded as invalid. Total efficacy rate = cured + markedly effective + effective.

### Statistical Analysis:

All the data were statistically analyzed by SPSS23.0 software, and the measurement data were expressed as (x̄±s) using t test. Counting data were expressed by percentage (%) using χ2 test; Rank sum test was used for ranked data. P<0.05 indicates a statistically significant difference.

## RESULTS

The total efficacy of the observation group was 87.5% after treatment, while that of the control group was 67.5%, with a statistically significant difference (*p*<0.05). Table-I.The TCM syndrome scores, local signs scores and pain scores of the two groups were significantly reduced after treatment (*p*<0.05), and the observation group showed a more significant reduction in the above scores (*p*<0.05). Table-II.The pelvic mass size and pelvic effusion of the two groups were significantly reduced after treatment (*p*<0.05), and the observation group showed a more significant reduction (*p*<0.05). Table-III.

**Table I:** Comparison of clinical efficacy between the two groups [case (%)].

Group	Number of cases	Cured	Markedly effective	Effective	Invalid	Total efficacy (%)
Control group	40	5(12.5)	10(25.0)	12(30.0)	13(32.5)	27(67.5)
Observation group	40	11(27.5)	16(40.0)	8(20.0)	5(12.5)	35(87.5)^*^

* *p*<0.05 compared with the control group.

**Table II:** Comparison of clinical index scores between the two groups before and after treatment (χ¯±scores).

Group	Time	n	TCM syndrome score	Local sign score	Pain score
Control group	Before treatment	40	14.23±2.65	9.12±1.13	4.81±0.52
	After treatment	40	7.13±0.83^*^	3.67±0.49^*^	2.91±0.40^*^
Observation group	Before treatment	40	14.16±2.54	9.04±1.08	4.75±0.56
	After treatment	40	2.87±0.41^*Δ^	1.94±0.33^*Δ^	1.40±0.27^*Δ^

* *p*<0.05 compared with this group before treatment;

Δ P<0.05 compared with the control group after treatment.

**Table III:** Comparison of pelvic mass size and pelvic effusion between the two groups before and after treatment (χ¯±S,mm).

Group	Time	n	Pelvic mass size	Pelvic effusion
Control group	Before treatment	40	31.18±4.28	43.25±5.53
	After treatment	40	19.32±3.17^*^	31.18±4.29^*^
Observation group	Before treatment	40	30.87±4.15	43.12±5.46
	After treatment	40	10.46±2.21^*Δ^	25.68±4.15^*Δ^

* *p*<0.05 compared with this group before treatment;

Δ *p*<0.05 compared with the control group after treatment

The PSV indexes of the two groups were significantly increased after treatment (*p*<0.05), and the observation group showed a more significant increase *(p*<0.05). Table-IV. The PI and RI indexes of the observation group were significantly reduced after treatment (*p*<0.05). There was no significant difference in the PI and RI indexed between the two groups before and after treatment (*p*>0.05).

**Table IV:** Comparison of uterine artery hemodynamic indexes between the two groups before and after treatment (χ¯±S).

Group	Time	n	PSV(cm/s)	PI	RI
Control group	Before treatment	40	27.23±3.89	2.55±0.37	0.86±0.11
	After treatment	40	29.59±4.08^*^	2.51±0.39	0.83±0.09
Observation group	Before treatment	40	26.87±4.12	2.60±0.41	0.85±0.09
	After treatment	40	32.18±4.46^*Δ^	2.17±0.34^*Δ^	0.75±0.08^*Δ^

* *p*<0.05 compared with this group before treatment;

Δ *p*<0.05 compared with the control group after treatment.

In the control group, four cases of nausea, three cases of diarrhea, one case of anorexia, one case of headache, two cases of rash occurred during the treatment period, with the incidence of adverse reactions of 27.5% (11/40). In the observation group, one case of stomach discomfort, one case of nausea and one case of anorexia occurred during the treatment, with the incidence of adverse reactions of 7.5% (3/40). There was a statistically significant difference in the incidence of adverse reactions between the two groups(*p*<0.05).

## DISCUSSION

SPID is a chronic inflammatory state that exists for a long time after pelvic infection, which is clinically characterized by adhesion, thickening and local extensive fibrosis of surrounding tissues. It is considered by modern medical research that the occurrence of SPID has a close bearing on menstrual sexual behavior, unclean sexual behavior, spread of peripheral organ infections and postoperative complications of gynecological diseases, and can be attributed to the ascending invasion and infection of microbial pathogens.^10^

According to traditional Chinese medicine, SPID belongs to heat invading the blood chamber, which is resemble the pathogenesis of diseases such as postpartum fever and leucorrhea. The author believes that the main pathogenesis of SPID is the accumulation of dampness and heat, blood stasis and stagnation, which will turn from excess to deficiency as the disease progresses, causing damage to the kidneys. Additionally, the uterine vessels are tied to the kidneys, and prolonged SPID illness will inevitably lead to physical weakness, resulting in diseases such as imbalance of Yin and Yang in the kidney or deficiency of kidney Qi, lumbossacral pain, infertility, menstruation disorder. Therefore, for the treatment of SPID, consideration should be given to clearing away heat and removing dampness, promoting blood circulation and removing blood stasis, and tonifying kidney. Significant efficacy in the treatment of SPID has been achieved by the “Fu Yan Qing prescription” formulated by the author. To formulate this prescription, Radix Paeoniae Rubra and Salvia miltiorrhiza are used as the principal drugs.^11^,^12^ With the combination of the two drugs, the function of promoting blood circulation, removing blood stasis, regulating menstruation and relieving pain can be exerted. Patrinia villosa Juss, Forsythiae Forsythiae, Rhizoma Smilacis Glabrae, Honeysuckle, Plantain Seed are used as the ministerial drugs.^13^-^15^ With the combination of Patrinia villosa Juss, Forsythiae Forsythiae and Honeysuckle, the function of detoxification and clearing heat can be exerted. Fructus Ligustri Lucidi, Rhizoma corydalis, Radix Dipsaci, Rhizoma Cyperi and Rhizoma Curcumae are used as the adjuvant and envoy drugs.^16^,^17^ These drugs can be used together to clear away heat and dehumidify, promote blood circulation and remove blood stasis, and nourish liver and kidney.

As shown in the results of this study, the TCM syndrome scores, local sign scores, VAS scores, pelvic mass and pelvic effusion volume in the observation group after treatment were significantly lower than those in the control group (*p*<0.05), indicating that Fu Yan Qing decoction can improve the local symptoms and signs of patients with SPID and relieve pelvic pain. The reason why Fu Yan Qing decoction can achieve such a favourable effect is that the traditional Chinese medicines such as Radix Paeoniae Rubra, Salvia miltiorrhiza, Rhizoma corydalis and Rhizoma Curcumae used in the prescription have the function of promoting blood circulation and removing blood stasis.^18^,^19^ The main pathological mechanism of PID has been proved to be that the formation of local inflammatory response is caused by the continuous stimulation of chronic inflammation, while the balance between the fibrinolytic system and the coagulation system is destroyed by repeated chronic inflammatory reaction, resulting in the increase of blood viscosity and the occurrence of pelvic blood circulation disorders.^20^

In this paper, patients in the two groups showed lower PSV levels, but higher PI and RI levels before treatment, suggesting that the blood viscosity of patients with SPID syndrome of accumulation of dampness heat and blood stasis type is increased, and pelvic blood is in a sticky state, which is consistent with the theory of “blood stasis” in traditional Chinese medicine. The PI and RI levels of the observation group are significantly decreased after treatment, while the PSV level is significantly increased, indicating that favourable effects can be achieved by Fu Yan Qing prescription in improving the pelvic blood stasis microenvironment of patients with SPID. After analysis, such a favourable effect has been proved to be closely linked to the effect of Fu Yan Qing prescription for promoting blood circulation and removing blood stasis. Moreover, the total efficacy of the observation group after treatment was significantly higher than that of the control group (*p*<0.05), and the incidence of adverse reactions was significantly lower than that of the control group (*p*<0.05), further indicating that the synergistic treatment effect can be exerted by the combination of antibiotics and Fu Yan Qing prescription in the treatment of PID, with a high drug safety.

### Limitations of the study:

It included small sample size, short follow-up time, and failure to divide and study the post-operative pathological types, therapeutic effects and prognosis of patients in a more detailed manner. We are actively increasing the sample size and further prolonging the follow-up time. Further clinical experiments are necessary.

## CONCLUSIONS

To put it in a nutshell, Fu Yan Qing prescription combined with antibiotics of western medicine is a safe and effective treatment regimen with significant synergistic effect. With such a regimen, significant benefits can be achieved in the treatment of SPID (accumulation of dampness heat and blood stasis type), such as promoting pelvic effusion and mass absorption, relieving pelvic pain symptoms, and improving pelvic circulation.

### Authors’ Contributions:

**SZ** & **LY:** Designed this study and prepared this manuscript, and are responsible and accountable for the accuracy or integrity of the work. **ZS:** Collected and analyzed clinical data. **YW:** Significantly revised this manuscript.

## References

[1] Curry A, Williams T, Penny ML (2019). Pelvic Inflammatory Disease:Diagnosis, Management, and Prevention. Am Fam Physician.

[2] Revzin MV, Mathur M, Dave HB, Macer ML, Spektor M (2016). Pelvic Inflammatory Disease:Multimodality Imaging Approach with Clinical-Pathologic Correlation. Radiographics.

[3] Chen Y, Wei S, Huang L, Luo M, Wu Y, Yin C (2020). Fuke Qianjin Combined with Antibiotic Therapy for Pelvic Inflammatory Disease:A Systematic Review and Meta-Analysis. Evid Based Complement Alternat Med.

[4] Zou W, Wen X, Zheng Y, Xiao Z, Luo J, Chen S (2015). Metabolomic Study on the Preventive Effect of Patrinia scabiosaefolia Fisch on Multipathogen Induced Pelvic Inflammatory Disease in Rats. Evid Based Complement Alternat Med.

[5] Yang LJ, Yang M, Miao RQ, Yu L, Li HR (2019). Discussion on opposing needling combined with dragon-tiger fighting needling for chronic pelvic inflammation. Zhongguo Zhen Jiu.

[6] Ma K, Luo SP, Li M, Zhang HX, Xu LM, Zhao RH (2017). Advantages and evidences research on Chinese medicine for treatment of pelvic inflammatory disease. Zhongguo Zhong Yao Za Zhi.

[7] Charveriat A, Fritel X (2019). Diagnosis of pelvic inflammatory disease:Clinical, paraclinical, imaging and laparoscopy criteria. CNGOF and SPILF Pelvic Inflammatory Diseases Guidelines. Gynecol Obstet Fertil Senol.

[8] Wang C, Chen J, Xiao Y, Shen Q (2020). Guizhi Fuling wan for chronic pelvic inflammatory disease protocol:A protocol for systematic review and meta-analysis. Medicine (Baltimore).

[9] Li Q, Chen CY, Suo YP, Huang M, Huang XH (2015). Evaluation on Efficacy and Safety of Jinying Capsule in Treatment of Pelvic Inflammatory Disease Patients with Accumulated Damp-heat Syndrome. Zhongguo Zhong Xi Yi Jie He Za Zhi.

[10] Wiringa AE, Ness RB, Darville T, Beigi RH, Haggerty CL (2020). Trichomonas vaginalis, endometritis and sequelae among women with clinically suspected pelvic inflammatory disease. Sex Transm Infect.

[11] Tan YQ, Chen HW, Li J, Wu QJ (2020). Efficacy, Chemical Constituents, and Pharmacological Actions of Radix Paeoniae Rubra and Radix Paeoniae Alba. Front Pharmacol.

[12] Wang L, Ma R, Liu C, Liu H, Zhu R, Guo S (2017). Salvia miltiorrhiza:A Potential Red Light to the Development of Cardiovascular Diseases. Curr Pharm Des.

[13] He X, Luan F, Zhao Z, Ning N, Li M, Jin L (2017). The Genus Patrinia:A Review of Traditional Uses, Phytochemical and Pharmacological Studies. Am J Chin Med.

[14] Liu R, Lai K, Xiao Y, Ren J (2017). Comparative pharmacokinetics of chlorogenic acid in beagles after oral administrations of single compound, the extracts of Lonicera japanica, and the mixture of chlorogenic acid, baicalin, and Forsythia suspense. Pharm Biol.

[15] Liang G, Nie Y, Chang Y, Zeng S, Liang C, Zheng X (2019). Protective effects of Rhizoma smilacis glabrae extracts on potassium oxonate- and monosodium urate-induced hyperuricemia and gout in mice. Phytomedicine.

[16] Zeng M, Li M, Zhang L, Zhang B, Wu G, Feng W (2019). Different meridian tropism in three Chinese medicines:Tinglizi (Semen Lepidii Apetali), Yiyiren (Semen Coicis), Cheqianzi (Semen Plantaginis). J Tradit Chin Med.

[17] Tian B, Tian M, Huang SM (2020). Advances in phytochemical and modern pharmacological research of Rhizoma Corydalis. Pharm Biol.

[18] Liu P, Shang EX, Zhu Y, Yu JG, Qian DW, Duan JA (2017). Comparative Analysis of Compatibility Effects on Invigorating Blood Circulation for Cyperi Rhizoma Series of Herb Pairs Using Untargeted Metabolomics. Front Pharmacol.

[19] Han H, Wang L, Liu Y, Shi X, Zhang X, Li M (2019). Combination of curcuma zedoary and kelp inhibits growth and metastasis of liver cancer in vivo and in vitro via reducing endogenous H2S levels. Food Funct.

[20] Sun H, Du XY, Wang HJ (2019). Research of the Influence on Uterine Hemodynamics Index and Oxidative Stress Levels of Jingangteng Tablets Combined with Cefoxitin Sodium Injection in Treating Patients with Sequelae of Pelvic Inflammatory Diseases. Chinese Sexual Sci.

